# Early History of the Saw

**Published:** 1890-09

**Authors:** 


### EARLY HISTORY OF THE SAW.

According to a recent writer, saws have been discovered in Germany and Denmark, which belong to the bronze age. The metal of which they were composed was cast into a thin shaft, and serrated by breaking the edge. Equally interesting discoveries have been made in America. It has been found that saws made of obsidian, which is a kind of glass produced by volcanoes, were used during the stone age in Mexico, and saws and knives of the same material have been found in the alluvial deposits of New Jersey, probably sent thither from Mexico by the action of the water. The Phœnicians are amongst the earliest nations which are supposed to have used the saw. The scholar is not surprised to find a very pretty story accounting for the discovery of the saw in Grecian mythology. Here the inventor is said to have found the jawbone of a snake, which he imitated by jagging an iron plate. The lacustrine and other early inhabitants of Europe are credited with having made saws of flint, and the natives of the West Indian islands had saws made of notched shells.

---

Medicinal preparations from the eucalyptus tree are coming into notice. The fluid extract is used in scalp and skin diseases, and has proved effectual in destroying the bacteria of erysipelatous affections.

